# Silencing of the *SlNAP7* gene influences plastid development and lycopene accumulation in tomato

**DOI:** 10.1038/srep38664

**Published:** 2016-12-08

**Authors:** Da-Qi Fu, Lan-Huan Meng, Ben-Zhong Zhu, Hong-Liang Zhu, Hua-Xue Yan, Yun-Bo Luo

**Affiliations:** 1 Laboratory of Fruit Biology, College of Food Science & Nutritional Engineering, China Agricultural University, Beijing, 100083, China; 2 Institute of Fruit Tree Research, Guangdong Academy of Agricultural Sciences, Guangzhou 510640, China; 3 Key Laboratory of South Subtropical Fruit Biology and Genetic Resource Utilisation, Ministry of Agriculture, Guangzhou 510640, China

## Abstract

Ripening is an important stage of fruit development. To screen the genes associated with pigment formation in tomato fruit, a suppression subtractive hybridization (*SSH*) cDNA library was constructed by using tomato fruit in the green ripe and break ripe stages, and 129 differential genes were obtained. Using redness as a screening marker, virus-induced gene silencing (VIGS) of the differential genes was performed with a sprout vacuum-infiltration system (SVI). The results showed that silencing the *SlNAP7* gene affected the chloroplast development of tomato leaves, manifesting as a photo-bleaching phenotype, and silenced fruit significantly affected the accumulation of lycopene, manifested as a yellow phenotype. In our study, we found that silencing the *SlNAP7* gene downregulates the expression of the *POR* and *PORA* genes and destroys the normal development of the chloroplast. The expression of related genes included in the lycopene biosynthesis pathway was not significantly changed, but lycopene accumulation was significantly reduced in tomato fruit. Perhaps it was caused by the destruction of the chromoplast, which leads to the oxidation of lycopene. The results show that the *SlNAP7* gene influences chloroplast development and lycopene accumulation in tomato.

Tomato (*Solanum lycopersicum*) fruit plays an important role in the human diet and provides health benefits as a source of vitamins, minerals, and antioxidants with substantial and growing economic and nutritional impact^1^. It is also an important model system for the study of fleshy fruit development and ripening due to its small genome, mature transgenic technology and complete genome information^2^. Fruit color formation is a typical feature of fruit ripening and an important factor for determining the quality of fruit. The change in tomato fruits from green to red is due to the degradation of chlorophyll and the accumulation of lycopene during the process of chloroplast-to-chromoplast conversion, which is normally regulated by some key ripening-associated genes during fruit ripening^3^. Several ripening-associated genes have been reported through the characterization of their mutants in tomato, including *rin* (ripening-inhibitor), *Cnr* (colorless non-ripening), *gr* (green-ripe), *gf* (green flesh), *hp* (high pigment), and *Nr* (never ripe)^4^,^5^,^6^,^7^,^8^,^9^, which affect fruit ripening and color formation. For example, tomato *lutescent1* and *2* mutants exhibit chlorophyll loss in leaves and fruits as well as a delay in fruit ripening^10^, whereas *high-pigment1, 2*, and *3 (hp1, hp2*, and *hp3*) mutants manifest increased levels of chlorophyll, carotenoids, and flavonoids compared with wild-type tomato^8^,^11^,^12^. Additionally, some studies have shown that the accumulation of carotenoids in tomato fruit is related to the number and structural integrity of fruit plastid. The suppression of *HY5*, for example, results in the loss of thylakoid organization and a decrease in carotenoid content^13^, whereas the suppression of the *CUL4* gene increases the plastid number, leading to lycopene accumulation in tomato fruit^14^. Furthermore, the over-expression of *APRR2-Like* increases the number and size of plastids, enhancing the chlorophyll and carotenoid levels in immature and red ripe tomato fruits, respectively^15^.

These key genes of fruit ripening affect the development of chloroplasts, which control fruit ripening, and other cofactors may also be involved in the process of fruit coloring and nutrition. Iron-sulfur (Fe-S) clusters are one example; these prosthetic groups play vital roles in major cellular processes, such as enzyme catalysis, electron transfer, and the regulation of gene expression, repair, and translation^16^,^17^,^18^,^19^,^20^. Independent Fe-S cluster assembly systems have been identified in the chloroplast (reviewed in Balk and Pilon^18^), with NFS2 (cysteine desulfurase) being the first Fe-S cluster assembly component identified in plastids^21^,^22^. Other Fe-S cluster components include *SufB*^23^; a point mutation of this gene in *Arabidopsis* leads to chlorophyll degradation and the accumulation of pheophorbide A^24^, and tobacco *SufB* mutants possess fewer chloroplasts. These data suggest that *SufB* is necessary for the assembly of Fe-S proteins to participate in chlorophyll metabolism^25^. In further support of their importance, data from *Arabidopsis* show that *SufC* and *SufD* mutants present an embryo-lethal phenotype^26^,^27^. Screening and identifying the cofactors involved in chloroplast development may help us understand fruit ripening.

To better understand the molecular regulation of tomato fruit development, we must try our best to screen and characterize all of the genes affecting the various aspects of fruit development, such as ripening, color, nutrition and so on. Although we can easily obtain a large number of candidate genes that may be involved in the development of tomato after tomato genome sequencing, our challenge is identifying the function of a large number of candidate genes in a short time. Traditional approaches such as mutant screening and plant transformation are often time consuming and labor intensive. In consideration of a longer life cycle and larger cultivation-area requirements compared with the model plant *Arabidopsis*, it is even more difficult to generate and maintain a saturated mutant population of tomatoes^28^. On the other hand, obtaining a transgenic plant is complex and not suitable for the functional characterization of a large number of genes. Virus-induced gene silencing (VIGS) technology will become an effective method of high-throughput functional identification of a large number of genes in plants because it is simple, fast and efficient, and it does not rely on genetic transformation, among other advantages^29^. For VIGS performance, we first cloned and inserted a fragment of the target gene into a virus vector to obtain a recombinant. The recombinant vector was then used to infect plants to trigger the post-transcriptional gene silencing (PTGS) of the plants, resulting in the silencing of endogenous genes homologous to the inserted fragment of the virus in the plant^30^,^31^.

In this study, we aimed to search for novel genes that influence the normal development of tomato fruit such as ripening, color, nutrition formation and so on by constructing and VIGS screening a suppression subtractive hybridization (SSH) library in tomato fruit. The results showed that the *SlNAP7* gene influences the plastid development and lycopene accumulation in tomato which contribute to the breeding of fruits with improved color.

## Results

### Construction of a suppression subtractive hybridization VIGS library

According to the changes in surface color during tomato fruit development, the ripening process can be divided into multiple stages, including immature (IM), mature green (MG), breaker (BR), turning (TU), and red ripening (RR)^32^. The specifically expressed genes in various stages are the key factors for determining the characteristics of each development stage, and they may be the key candidates influencing fruit development and ripening. At the same time, the expression levels of the same gene may be different at different stages^33^. To screen some candidate genes involved in the conversion of the fruit development stage, MG-stage and BR-stage tomato fruit were selected as the driver and the tester to construct a suppression subtractive hybridization (SSH) screen because the two-stage conversion is the key color change process during fruit ripening. Tobacco rattle viral vector TRV2 was used to replace the clone vector of the commercial SSH kit; the differential expression cDNA, which was more highly expressed in the BR stage than in the MG stage at the onset of ripening, was successfully inserted into the TRV2 vector to form the VIGS cDNA library. After the clone sequencing, we obtained 129 clones with independent sequences from 850 clones (Supplemental DataS1). Data have shown that these clones are involved in ethylene biosynthesis and signal transduction (*SlACO1, SlACO4*), lycopene synthesis (*PSY1*), softening (*PL*) and ripening–associated transcription factors (*TDR4, AP2a* and *Rin*). The sequencing results of the SSH library showed that it could be used as a resource to screen new key genes influencing tomato fruit development.

### VIGS screen identified a SufC-like ATP-binding cassette/ATPase gene influencing fruit development

**Figure 1:**
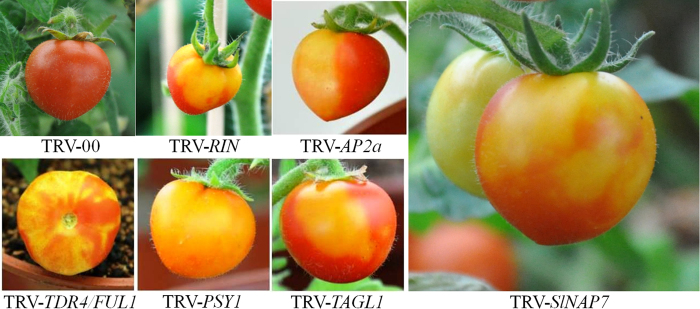
Phenotypes of *SlNAP7, RIN, AP2a, TDR4/FUL1, PSY1*, and *TAGL1*-silenced fruits of‘Micro-Tom’. TRV-00 fruit is the control.

To obtain new genes influencing fruit development from the SSH cDNA library, a mixture of *Agrobacterium* GV3101 cultures containing pTRV1 and pTRV2-candidate constructs (129 clones) in a 1:1 ratio were vacuum-infected in 0.5–1 cm sprouts of Micro-tom tomato. The sprouts infiltrated with *Agrobacterium* cultures including pTRV1 and pTRV2 or pTRV2-*PDS* were used as a negative or positive control. These infected sprouts were planted in the soil to maintain growth under suitable conditions, and the development of seedling and fruits with candidate gene silencing was observed and recorded. Approximately 35–40 days after pollination, six clones infected plants produced some defectively ripening fruit showing red and yellow sections, whereas all control plants injected with the pTRV-empty vector produced normally ripening fruit. Clone sequencing indicated that 5 of the 6 clones have been reported to influence tomato fruit ripening including *RIN, AP2a, TDR4/FUL1, PSY1*, and *TAGL1* (Fig. 1). All non- or delayed-ripening phenotypes of the 6 gene-silencing treatments were represented by more than 10 plants across two or more independent experiments; >30 fruits per gene exhibited the phenotype depicted in Fig. 1. The results showed that VIGS is a rapid and effective method for the screening of the important genes affecting fruit development and ripening.

In addition to the five genes above reported, we also identified a clone (No.107) with a novel gene that potentially affects lycopene accumulation in tomato fruit (Fig. 1). The sequence analysis with BLAST on Phytozome 10.3 (http://phytozome.jgi.doe.gov) revealed that clone 107 contains a cDNA fragment of the tomato gene Solyc06g048540.2.1, which encodes a SufC-like ATP-binding cassette/ATPase. The phylogenetic analysis indicated that this novel gene is closest to the *Arabidopsis* homologue *AtNAP7*, which plays an essential role during embryo genesis (Supplemental DataS2)^26^. We named the Solyc06g048540.2.1 gene *SlNAP7*.

### *SlNAP7* is upregulated during normal ripening and downregulated in non-ripening mutants

**Figure 2:**
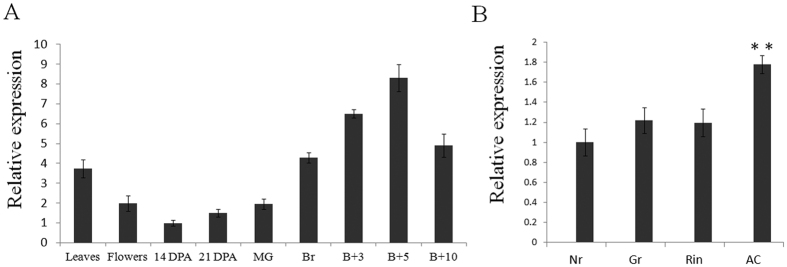
*SlNAP7* expression in different wild-type and mutant tissues across various fruit developmental stages. (**A**) qRT-PCR expression analysis of *SlNAP7* in leaves, flowers, and fruits of various developmental stages (14 DPA, 14 days post-anthesis; 21 DPA, 21 days post-anthesis; MG, mature green; BR, breaker; B+3, 3 days after breaker; B + 10, 10 days after breaker) in ‘*Ailsa Craig*’ wild-type tomato. (**B**) qRT-PCR expression analysis of *SlNAP7* in *Nr, Gr, Rin* mutants and ‘Ailsa Craig’ wild-type tomato at the breaker stage. Asterisks indicate significant differences (Student’s *t*-test, **P < 0.05).

To study the role of *SlNAP7* during tomato development, we measured its expression level by quantitative real-time PCR (qRT-PCR) analysis. Several tissues and developmental states of ‘*Ailsa Craig*’ tomatoes were tested, including leaves, flowers, and fruits at different stages (14 days post-anthesis [DPA]; 21 DPA; MG; BR; 3 days after BR; RR or 7 days after BR; 10 days after BR) (Fig. 2A). Our data showed that the expression level of the *SlNAP7* gene was relatively low during the immature stages but strongly upregulated between the mature green and breaker stages. The amount of *SlNAP7* mRNA transcripts in the breaker stage was nearly 2.5-fold higher than the amount in the pre-mature green stages; the peak was reached at the RR stage. To further investigate whether a relationship existed between *SlNAP7* and ripening, we analyzed its expression in fruit-ripening mutants, including *rin, Gr*, and *Nr*^4^,^6^,^9^. The qRT-PCR results revealed that *SlNAP7* gene expression was downregulated by approximately 40% in mutants compared with wild-type tomato (Fig. 2B), suggesting that *SlNAP7* plays a role during tomato fruit development.

### SlNAP7-GFP is targeted to the plastids in tobacco mesophyll protoplasts

**Figure 3:**
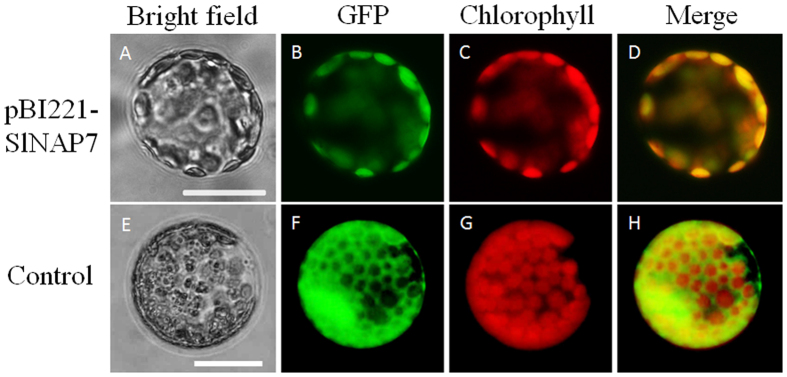
Subcellular localization of a SlNAP7 protein in tobacco mesophyll protoplasts. (**A,E**) Bright field. (**B,F**) Green fluorescence (from GFP) in tobacco mesophyll protoplasts when excited with UV light. Protoplasts were transfected with the plasmids pBI2211 and pBI221-*SlNAP7* with GFP gene. (**C,G**) Red chlorophyll fluorescence induced with ultraviolet light. (**D**) Merged fluorescent signals of (**B** and **C**). (**H**) Merged fluorescent signals of f and g.

To examine the sub-cellular localization of SlNAP7 *in vivo*, we fused the *SlNAP7* coding region in-frame with the N-terminal side of the green fluorescent protein (GFP) under the control of the CaMV 35 S promoter. The SlNAP7-GFP and GFP control plasmids were transiently expressed in tobacco leaves by *Agrobacterium* infiltration. While control GFP accumulated in both the nucleus and cytoplasm, the SlNAP7-GFP fusion protein was clearly localized in the chloroplast (Fig. 3). This suggests that SlNAP7 is a chloroplast-targeting protein (Fig. 3). Xu *et al*. (2004) performed a subcellular localization of *Arabidopsis* NAP7 protein in tobacco cells. The result also showed that the AtNAP7 protein was located in the chloroplast membrane^26^.

### VIGS of *SlNAP7* affects chlorophyll accumulation and thylakoid structure in leaf

**Figure 4:**
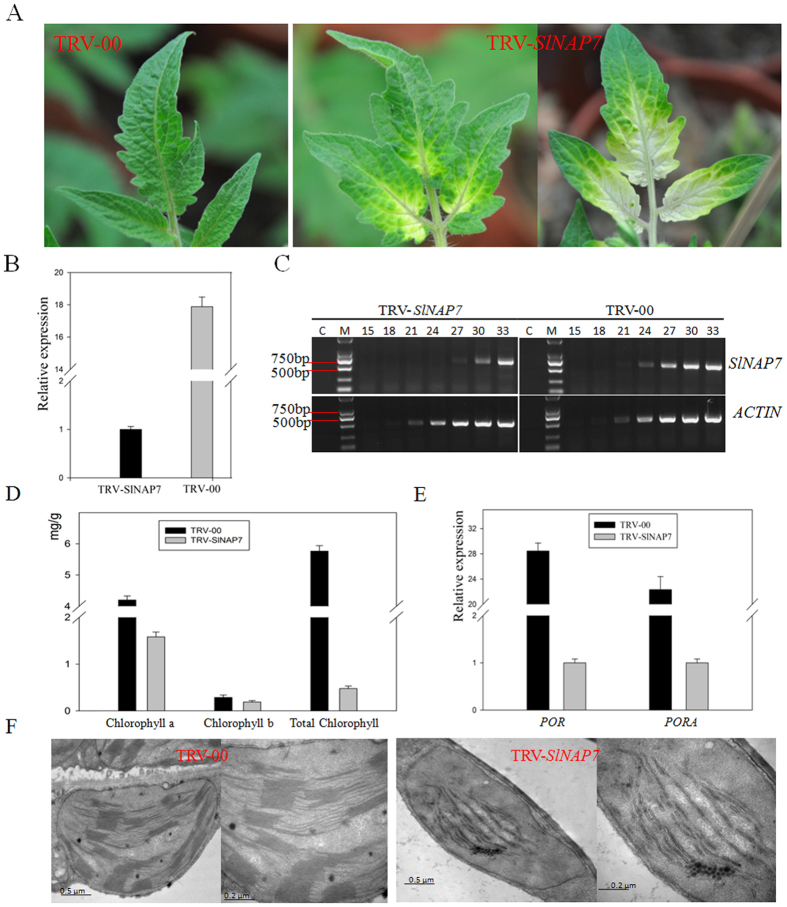
A virus-induced gene silencing (VIGS) construct in tomato seedlings reveals the effect of *SlNAP7* on chlorophyll accumulation. (**A**) The white phenotype on leaves and seedlings indicates efficient silencing of *SlNAP7*. (**B,C**) qRT-PCR and semi-quantitative RT-PCR expression analysis of *SlNAP7* in control (TRV-00) and *SlNAP7*-silenced tomato leaves. The PCR cycle number is indicated above the lanes. M is the DNA size marker (Takara DL2000 DNA marker), and C is the no-RT control. *ACTIN* is the internal standard/loading control. *SlNAP7* mRNA decreased significantly in *SlNAP7*-silenced leaves compared with the control. (**D**) Chlorophyll a, chlorophyll b, and total chlorophyll content in leaves of control and *SlNAP7*-silenced plants. (**E**) Relative expression of *POR* and *PORA* in control and *SlNAP7*-silenced plants. (**F**) Transmission electron microscopy of tomato leaf chloroplasts in controland*SlNAP7*-silenced plants. Typical thylakoids including grana stacks can be observed in control leaves (TRV-00), whereas *SlNAP7*-silenced leaves exhibit deficiencies in thylakoid organization.

To test the function of *SlNAP7* in the chloroplast, we used VIGS to silence *SlNAP7* in tomato leaves. Tomato plants were infiltrated with *Agrobacterium* containing either pTRV2-*SlNAP7* and pTRV1 or pTRV1 and pTRV2 alone (empty vectors) as a control. The results showed that the *SlNAP7*-silenced leaves were photo-bleached compared with the TRV-infected control plants (Fig. 4A). We examined the gene expression of *SlNAP7* in the silenced plants using TRV-infected plants as a negative control. The *SlNAP7* transcripts in the photo-bleached leaves showed ~95% downregulation when compared with the control (Fig. 4B and C), confirming that the photo-bleaching is due to the silencing of *SlNAP7*. We noted that the *SlNAP7*-silenced tomato leaf failed to accumulate chlorophyll (Fig. 4A); in VIGS leaves, the chlorophyll a, chlorophyll b, and total chlorophyll content were reduced by 93%, 88%, and 92%, respectively (Fig. 4D).

To explain why silencing the *SlNAP7* gene leads to the degradation of chlorophyll in tomato leaves at the molecular level, the expression of 20 genes associated with chlorophyll metabolism was detected by qRT-PCR with the *Actin* gene as the inner control (for all primer sequences, see Supplemental Data S3). The results showed that the expression of NADPH: protochlorophyllide oxidoreductase (*POR*) and NADPH: protochlorophyllide oxidoreductase A (*PORA*) OPRA and OPRB genes were significantly downregulated by 90% and 95%, respectively, in *SlNAP7*-silenced leaves compared with control leaves (Fig. 4E). There was no significant difference in the expression of the other 18 genes between the two samples above. To further detect whether *SlNAP7* silencing affected the development of chloroplasts, transmission electron microscopy was used to observe the status of leaf plastids in the leaves of *SlNAP7*-silenced and control tomatoes. The results indicate that the *SlNAP7*-silenced plants had clear deficiencies in both thylakoid organization and abundance (Fig. 4F), suggesting that *SlNAP7* gene silencing decreased chlorophyll accumulation in part through a disruption of plastid structure. In conclusion, SlNAP7 protein is required for chloroplast development in tomato leaves, and it influences the accumulation of chlorophyll.

### VIGS *SlNAP7* influences tomato flower and fruit pigmentation

**Figure 5:**
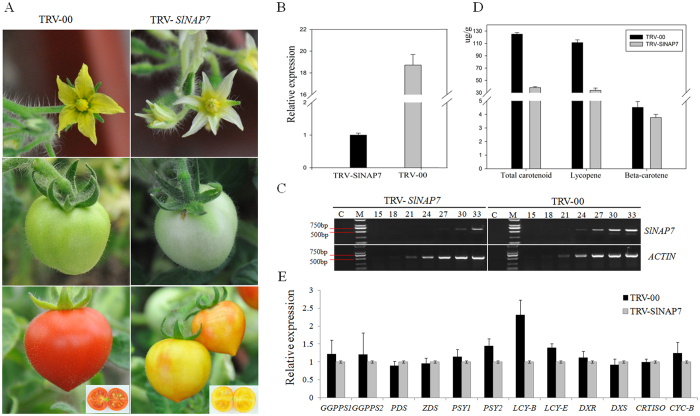
Phenotypes of flower and fruit from the *SlNAP7*-silenced ‘Micro-Tom’ plant. (**A**) Flowers and fruits (mature green and B+10) of the control (TRV-00; left) and *SlNAP7* (right). (**B, C**) qRT-PCR and semi-quantitative RT-PCR expression analysis of *SlNAP7* in control (TRV-00) and *SlNAP7*-silenced tomato fruits. The PCR cycle number is indicated above the lanes. M is the DNA size marker (Takara DL2000 DNA marker), and C is the no-RT control. *ACTIN* is the internal standard/loading control. *SlNAP7* mRNA significantly decreased in *SlNAP7*-silenced fruits compared with the control. (**D**) Comparison of total carotenoid, lycopene, and β-carotene content in control (TRV-00) and *SlNAP7*-silenced tomato fruits at stage B + 10. (**E**) Relative transcript level of genes associated with the carotenoid biosynthesis pathway in control (TRV-00) and *SlNAP7*-silenced tomato at the breaker stage.

In *SlNAP7*-silenced tomato plants, normal pigment accumulation was affected in reproductive organs, including flowers and fruits at different developmental stages. Compared with the yellow petals in control plants, *SlNAP7*-silenced flowers exhibited an obvious white petal phenotype (Fig. 5A). White petals were also observed in immature and mature green fruits, implying that the phenotype is due to systemic silencing of the *SlNAP7* gene (Fig. 5A). Ripe fruits of the *SlNAP7*-silenced plants did not turn red upon ripening and exhibited a yellow color on the pericarp and pulp when the silenced fruit was cut (Fig. 5A).

To confirm these morphological results at the molecular level, we used semi-qRT-PCR and qRT-PCR to measure the *SlNAP7* transcript levels in the control and *SlNAP7-*silenced fruits at the same developmental stage (3 days after BR). Compared with the control red fruit, the level of *SlNAP7* transcripts in the yellow fruit were downregulated by nearly 95% (Fig. 5B,C). These results suggest that the yellow tomato fruit phenotype was due to the silencing of *SlNAP7*.

The ripening of tomato fruit is associated with a dramatic increase in lycopene content. To verify whether the yellow phenotype of *SlNAP7*-silenced fruit is related to the accumulation of lycopene, the lycopene and its metabolites were analyzed by High Performance Liquid Chromatography (HPLC) at the RR stage of the *SlNAP7*-silenced and control fruits. The data show that the total carotenoid, lycopene, and β-carotene contents were reduced by approximately 69.2%, 69.6%, and 16.5%, respectively (Fig. 5D) in *SlNAP7*-silenced fruit compared with in control fruit. To further investigate whether the low pigment content was due to a decrease in the expression of genes associated with carotenoid biosynthesis, we used qRT-PCR to examine the expression of *GGPPS1, GGPPS2, PDS, ZDS, PSY1, PSY2, LCY-B, LCY-E, DXR, DXS, CRTISO*, and *CYC-B*^34^,^35^. The results revealed that the expression of these genes in the *SlNAP7*-silenced tomato fruit was not significantly different compared with the control (Fig. 5E), suggesting that *SlNAP7* does not directly affect the synthesis of lycopene but rather may affect lycopene stability in tomato fruit.

## Discussion

Subtractive cDNA hybridization is a powerful tool for the screening and cloning of novel genes differentially expressed across two tissues or experimental treatments^36^. However, while the resultant SSH cDNA library can generate many candidate genes, it remains a challenge for researchers to assign gene functions comprehensively. In this study, we constructed a SSH-VIGS cDNA library, generating 129 genes expressed differently between BR and MG tomatoes, a model fruit ideal for understanding climacteric ripening mechanisms. We addressed the difficulties of gene characterization by screening the library using SVI^37^. As a result, we were able to identify the silenced phenotypes of major genes that regulate tomato fruit ripening, including *RIN, AP2a, TDR4/FUL1, PSY1*, and *TAGL1* (Fig. 1). Moreover, the VIGS screen allowed us to observe for the first time leaf photo-bleaching and non-ripening fruit phenotypes of *SlNAP7* silencing. Our results demonstrated the usefulness of VIGS in characterizing genes for which knockout mutants might be difficult to obtain^38^,^39^.

Here, we found that silencing *SlNAP7* disrupted chlorophyll accumulation in the leaves and immature fruits, with photo-damaged spots observed even under ambient lighting conditions. Additionally, our expression analysis revealed that *SlNAP7* likely acts upstream of other major genes involved in chlorophyll biosynthesis and accumulation, including light-dependent NADPH:PORA or NADPH:PORB^40^, because both *POR* and *PORA* transcript levels were downregulated in *SlNAP7*-silenced leaves.

Related to its role in chlorophyll accumulation, *SlNAP7* appears to be involved in the color change that occurs when tomato fruits ripen, as *SlNAP7*-silenced fruits exhibited a yellow phenotype instead of turning red. Because the red color in ripe tomato fruits is due to the accumulation of all-trans-lycopene (a carotenoid produced during fruit ripening^41^,^42^) and we observed an overall decrease in total carotenoid and lycopene content, *SlNAP7* likely functions in the carotenoid metabolic pathway. However, we found no significant differences between *SlNAP7*-silenced mutants and wild-type controls in the expression of genes related to carotenoid biosynthesis and accumulation. These genes include *PSY1*, which encodes phytoene synthase, an enzyme that catalyzes the rate-limiting step (phytoene formation from geranylgeranyl diphosphate) at the start of carotenoid biosynthesis^43^,^44^. Previous research has demonstrated that the inhibition of *PSY1* expression causes a failure to accumulate lycopene in mutant fruits^45^,^46^. Our data, however, suggest that the decrease in pigment concentration is not primarily due to changes in the expression of *PSY1* and similar genes.

Instead, we believe that the yellow phenotype of *SlNAP7*-silenced mutants was due to a disruption in typical plastid development. The transformation of chloroplasts to lycopene-accumulating chromoplasts is a hallmark of tomato fruit ripening^47^, and tomato *hp1, hp2*, and *hp3* mutants, as well as plants with an *APRR2*-like over-expression, exhibit higher carotenoid content along with an increase in chromoplast number or area^8^,^11^,^12^,^13^,^15^. In our study, *SlNAP7* expression during the post-BR stage is upregulated in wild-type tomatoes and downregulated in non-ripening mutants, suggesting that the gene is critical in plastid development during climacteric fruit ripening.

## Materials and Methods

### Plant materials

Wild-type tomato (Wt; *S. lycopersicum*‘*Ailsa Craig*’) and its near-isogenic lines containing *rin (rin/rin*), *Nr*, and *Gr* non-ripening mutants were grown in a temperature-controlled greenhouse. For the measurement of the time until ripening, flowers were tagged at anthesis, and fruit development was recorded as DPA. Fruits were harvested at the following stages: 14 DPA, 21 DPA, MG (approximately 40 DPA), BR (approximately 41–42 DPA), B+3 (3 days after BR), B+7 (7 days after BR), B + 10 (10 days after BR)^32^. The ripening stages of the non-ripening cultivars *rin, Nr*, and *Gr* were calculated based on DPA using wild-type tomato as a reference.

The VIGS experiments were performed using the wild-type variety *S. lycopersicum* ‘*Micro Tom*’. Seeds were germinated in flasks containing sterile water. Once the germinating seeds reached 0.5–1 cm in length, they were subjected to sprout vacuum-infiltration^37^. The treated sprouts were sown in 10 cm-diameter pots and housed in an environment-controlled growth room (23 ± 2 °C, 50–70% relative humidity, RH) under 16 h light/8 h dark and 250 mol/m^2^/s light. Fertilizer, including a soluble trace element mix, was applied daily with water.

### SSH-VIGS library construction

We performed PCR-based subtractive cDNA library construction following the manufacturer’s protocol (Clontech, Cat. 637401) with some modifications. Total RNA was isolated from (driver) and BR (tester) tomato fruits according to CTAB methods^48^. We followed the protocol included in the Oligotex mRNA Mini Kit (Qiagen, Cat. No. 70022) to purify mRNA from the total isolated RNA. We then examined the RNA integrity with 2% agarose gel electrophoresis.

Double-stranded cDNA was synthesized from 5 μg of the purified driver and tester poly (A)^+^ RNA, each in 20-μL reaction solutions, using the M-MLV RTase cDNA Synthesis Kit (Takara D6130). The dscDNA was digested separately with *Rsa*I (New England Biolabs, NEB) to acquire cDNA fragments of an appropriate size for VIGS. The cDNA was then phenol-extracted and ethanol-precipitated. The digested driver and tester cDNA was re-suspended in 10 μL of double-distilled sterile water. One microliter of the *Rsa*I-digested cDNA from the BR tomato fruits was diluted in 5 μL H_2_O and divided into two equal parts. The cDNA was then ligated to adapter 1 and adapter 2 R (10 μM) in separate ligation reactions. Two rounds of hybridization were performed. The final solution was diluted and mixed in 200 μL of dilution buffer (20 mM Hepes, pH 8.3/50 mM NaCl/0.2 mM EDTA), heated at 72 °C for 7 min, and stored at −20 °C.

To reduce background noise and enrich differentially expressed sequences, two rounds of PCR were conducted with two different primer sets (primer P1 for the primary PCR, nested-PCR primer1 with an *EcoR*I site and nested-PCR primer 2R with a *BamH*I site for the secondary PCR) under the same conditions. The PCR products were digested with the restriction endonucleases *EcoR*I and *BamH*I and inserted into the respective restriction sites of the cleaved TRV-RNA2 VIGS vector, pYL156. Subsequently, the vector was transformed into *E. coli* for amplification. Plasmids were isolated from bacterial cells and quantified for introduction into *A. tumefaciens* (GV3101) via electroporation.

### Sprout vacuum-infiltration

Sprout vacuum-infiltration was performed following the methods of Yan *et al*.^37^. A single colony (*Agrobacterium* strain containing TRV1, TRV2 alone, or TRV-SSH) was selected and then inoculated in 2 mL of liquid LB medium via shaking at 200 rpm and 28 °C for 10 h. Next, 500 μL of culture was transferred to clearly labelled Falcon tubes containing 3 mL of LB broth with the appropriate antibiotics, 10 mM MES and 20 μM acetosyringone, and then incubated with shaking at 200 rpm and 28 °C for 10–12 h. After incubation, we mixed each *Agrobacterium* culture (one *Agrobacterium* strain containing TRV1 and the other strain selected from the SSH-VIGS cDNA library) in a 1:1 ratio by volume. The bacterial cells were harvested via centrifugation at 5,000 × *g* for 5 min, re-suspended in 3 mL of infiltration buffer (10 mM MgCl_2_, 10 mM MES, 200 μM acetosyringone; pH 5.6), and left at room temperature for 2–3 h. Upon diluting the solution to 0.05% (v/v), Silwet L77 was added and mixed immediately. The *Agrobacterium* solution was infiltrated into the sprouts using SVI with a relative vacuum pressure of −25 kPa. Vacuum pressure was maintained for approximately 20 s and then released rapidly until atmospheric pressure was reached. The procedure was repeated twice, and the sprouts were subsequently sowed.

### Determination of chlorophyll content

We ground 0.2–0.5 g of leaf tissue from *SlNAP7*-silenced and control plants to a fine powder in liquid nitrogen, and then added 30 mL of 80% acetone (v/v) for extraction. The tissue homogenate was centrifuged at 4000 ×*g* and 4 °C for 10 min. The supernatant was separated and used for the chlorophyll assay.

Three independent replicates of the samples were analyzed. The sample absorbance was immediately measured in a DR3900UV-visible spectrophotometer (Hach Co.) at 645 nm and 663 nm using a 1-cm path length curette. The values were accurate to three decimal places. Chlorophyll contents were calculated with the following equations and expressed as mg g^−1^ fresh weight (FW):


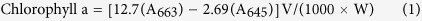



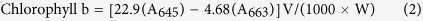



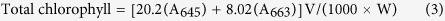


where A645 and A_663_ = extinction coefficients at their respective wavelengths, V = volume of the extract (mL), and W = weight of fresh leaves (g).

### Carotenoid extraction and analysis

The extraction and high-performance liquid chromatography (HPLC) separation of carotenoids were performed as previously described^49^. The freeze-dried tomato fruit pericarp (from 8–10 each of *SlNAP7*-silenced and control fruits) was ground into powder in liquid nitrogen. We then added 0.5–1 g of the powdered samples into 14-mL centrifugal tubes (Corning) and mixed with methanol (1.5 mL) via inversion for 5 min at 4 °C. We added 1.5 mL of Tris-HCl (50 mM, pH7.5, containing 1 M NaCl) and incubated the solution for 10 min at 4 °C. Next, we added 4 mL of chloroform and incubated the mixture on ice for another 10 min. Carotenoids were partitioned into chloroform using centrifugation, after which the organic phase was removed and the aqueous phase was re-extracted with chloroform. Finally, the extract was evaporated to near dryness under a nitrogen stream.

To determine the total carotenoid content, the sample absorbance was immediately measured in a DR3900UV-visible spectrophotometer (Hach Co.) at 425 nm. The total carotenoid content (μg ml^−1^) was calculated with equation (4): E_450_/0.25.

The separation of lycopene and *β*-carotene using HPLC was performed on an Agilent 1260 system (Agilent Technologies) using a C18 reverse-phase column (250 mm×4.6 mm, 5 μm) purchased from YMC. The extract was dissolved in dichloromethane and diluted to a constant volume; the concentration was adjusted during the mobile phase. Lycopene and *β*-carotene were identified via their characteristic absorption spectra, the typical retention time, and a comparison with authentic standards. The HPLC grade *β*-carotene (L9879) and lycopene (C4582) standards were obtained from Sigma. The mobile-phase composition was methanol: methyl cyanide: dichloromethane (7:7:2), and the flow rate was 1.2 mL/min. Detection was performed at 475 nm for lycopene and 425 nm for β-carotene with an online photodiode array detector (Agilent 1260 Infinity LC).

### Measurement of mRNA with qRT-PCR and semi-qRT-PCR

Tomato fruit pericarp (from 8–10 tomato fruits) or leaves were ground to a fine powder in liquid nitrogen and stored at −80 °C. We isolated total RNA from fruits and leaves following CTAB methods (http://www.arabidopsisthaliana.com/CTABRNA.htm). Total RNA was quantified using a NanoDrop 2000 spectrometer (Thermo Scientific) and fractionated on 1% (w/v) agarose gels. Next, the RNA was treated with RNase-free DNaseI (Promega) and reverse-transcribed with M-MLV reverse transcriptase (Promega). We used 1 mL of the resultant cDNA solution for the qRT-PCR analysis.

Quantitative real-time PCR was performed using iQ SYBR Green Supermix (Bio-Rad) on a CFX 96 Real-time Thermal Cycler system. Relative quantification of mRNA was performed using the comparative Ct (2^−ddCt^) method in the Bio-Rad CFX Manager software bundled with the thermal cycler. Next, semi-qRT-PCR was performed in a T-Gradient Thermocycler (Biometra Ltd.), and the products were examined on 1% (w/v) agarose gels. Three biological replicates for each sample were analyzed, and standard curves were run simultaneously. A tomato *ACTIN* gene (GenBank no. AB199316–1) was used as the internal standard. All gene-specific primers for PCR amplification are listed in Supplemental Table S1.

### Transmission electron microscopy

Leaves (*SlNAP7*-silenced and control, approximately 1 mm^2^) were fixed using a mixture of 2.5% glutaraldehyde and 2.5% paraformaldehyde in 0.1 M sodium cacodylate buffer (pH6.8) at 4 °C for 24 h. The samples were then fixed with 1% osmium tetroxide at 4 °C and dehydrated in a graded alcohol series. The samples were infiltrated and embedded in Poly/Bed 812 resin (Polysciences, Inc.). We used a PTXL ultramicrotome (RMC, Boeckeler Instruments) to obtain thin tissue sections (70-nm thickness) that were stained with uranyl acetate and lead citrate on 200-mesh copper grids. The sections were imaged using an H-7650B transmission electron microscope (Hitachi, Ltd.) at an accelerating voltage of 80.0 kV.

### Statistical analyses

The data were analyzed and graphed in Sigma Plot for Windows (version 11.0, Systat Software, Inc., Erkrath, Germany). One-way ANOVA was used to analyze group differences in gene expression. Student’s *t*-tests were used to compare two means. Statistical significance was set at P < 0.05. All data are presented as the means of three biological replicates ± standard error. Error bars represent the standard error of the mean (SEM), n = 3.

## Additional Information

**How to cite this article**: Fu, D.-Q. *et al*. Silencing of the *SlNAP7* gene influences plastid development and lycopene accumulation in tomato. *Sci. Rep.* **6**, 38664; doi: 10.1038/srep38664 (2016).

**Publisher's note:** Springer Nature remains neutral with regard to jurisdictional claims in published maps and institutional affiliations.

## Supplementary Material

Supplementary Information
