# Supplementary material for: Silencing of the SlNAP7 gene influences plastid development and lycopene accumulation in tomato
Source: Sci Rep. 2016 Dec 8;6:38664. doi: 10.1038/srep38664 (PMC5144135; doi:10.1038/srep38664)
Supplement: Supplementary Information [file srep38664-s1.pdf]

Silencing of the *SlNAP7* gene influences plastid development and lycopene accumulation in tomato

Da-Qi Fu[1], Lan-Huan Meng [1], Ben-Zhong, Zhu [1], Honaliang Zhu[1],

Hua-Xue, Yan[2, 3*], Yun-Bo Luo[1*]

1. Laboratory of Fruit Biology, College of Food Science & Nutritional Engineering, China Agricultural University, Beijing，100083, China

2. Institute of Fruit Tree Research, Guangdong Academy of Agricultural Sciences, Guangzhou, 510642, China

3. Key Laboratory of South Subtropical Fruit Biology and Genetic Resource Utilization, Ministry of Agriculture, Guangzhou 510640, China

*Correspondence should be addressed to Hua-Xue Yan (ylp840203@163.com) and Yun-Bo Luo (lyb@cau.edu.cn).

Supplemental Data 1. Statistical analysis of the cDNA sequencing results of the SSH-VIGS cDNA library.

No.	Accession	Description
1	GU784870.1	fructan beta-fructosidase/ Tomato acid invertase (TIV1)
2	HQ322499.1	*SlACO1*
3	NM_001246999.1	*SlACO4*
4	AK247917.1	Chitin-binding lectin 1 precursor
5	NM_001247898.1	Catalase isozyme 1
6	AK328001.1	Wound-induced protein WIN2 precursor
7	NM_001247102.1	Superoxide dismutase [Cu-Zn] 2
8	AK328092.1	1-aminocyclopropane-1-carboxylate oxidase (E8) homolog
9	NM_001247833.1	fruit-specific protein; Metallocarboxypeptidase inhibitor
10	AK319988.1	Aluminum-induced protein
11	AK319919.1	Elongation factor 1-alpha
12	AK325382.1	60S ribosomal protein L12
13	AK324984.1	P40-like protein
14	AK322550.1	UDP-glycosyltransferase 73E1
15	AK321129.1	Chromosome chr8 scaffold_99
16	AK323015.1	Late embryogenis abundant protein 5
17	AK246436.1	GRF zinc finger family protein
18	AK324180.1	Alcohol dehydrogenase
19	AK329721.1	Putative calcyclin-binding protein
20	AK324307.1	Ribosomal protein S8
21	AK224673.2	Hypoxia-responsive family protein
22	AK324615.1	Chromosome chr5 scaffold_64
23	AK327019.1	Glyoxysomal beta-ketoacyl-thiolase
24	AK321378.1	Putative uncharacterized protein
25	AK329816.1	Chromosome chr8 scaffold_34
26	AK322751.1	Putative uncharacterized protein
27	EU734550.1	Phytoene synthase 1 (PSY1)
28	AK323048.1	Flowering locus T-like 1 protein/ Metallothionein-like protein type 2 B
29	NM_001247637.1	Translationally-controlled tumor protein homolog / P23-like protein
30	NM_001247092.1	Polygalacturonase-2 precursor
31	NM_001247113.1	Photosystem II 10 kDa polypeptide
32	NM_001247210.1	plasma membrane intrinsic protein 1C
33	NM_001247083.1	S-adenosyl-l-homocysteine hydrolase (SAHH) / Adenosylhomocysteinase
34	AF123255.1	17.7 kD class I small heat shock protein
35	AK319828.1	AP2/ERF domain-containing transcription factor(*AP2a*)
36	XM_002330021.1	Putative uncharacterized protein
37	DQ191661.1	60s ribosomal protein L27a-like protein
38	AK319555.1	Ribulose bisphosphate carboxylase small chain 2A
39	AK326205.1	Cytochrome P450-dependent fatty acid hydroxylase
40	AK323478.1	40S ribosomal protein S26 (RPS26C)

41	NM_001247150.1	Phosphoenolpyruvate carboxykinase
42	NM_001247567.1	Alcohol acyl transferase
43	AK224647.2	Putative uncharacterized protein
44	NM_001247300.1	BTF3-like transcription factor
45	AK326576.1	2-isopropylmalate synthase A
46	NM_001247244.1	TDR4 transcription factor
47	AK327909.1	PREDICTED: Solanum lycopersicum stem-specific protein TSJT1-like
48	EU122386.1	KDEL-tailed cysteine endopeptidase
49	JN033214.1	methionine sulfoxide reductase A (MSRA)
50	AK326232.1	Actin-depolymerizing factor
51	AK323947.1	Putative uncharacterized protein, homologue to 40S ribosomal protein S23
52	AK224882.2	28 kDa small subunit ribosomal protein
53	AK247694.1	NADP-isocitrate dehydrogenase
54	AK224781.2	Putative uncharacterized protein
55	AK246381.1	Ribosomal protein L24-like protein
56	TC232632	ABC transporter-like protein
57	TC238301	Putative MAWD binding protein
58	NM_001247741.1	MADS-box transcription factor MADS-RIN
59	AK326001.1	Abscisic stress-ripening protein 1 (ASR1)
60	AF096261.1	Methionine synthase (MS)
61	BT014320.1	chloroplast tRNA-Ala tRNA-Ile 16S rRNA tRNA-Val
62	BT013193.1	Ankyrin-repeat protein HBP1
63	AK327244.1	Putative cytidine deaminase
64	AK324910.1	ADP/ATP translocator-like
65	AK246696.1	Ribosomal protein L37
66	NM_001247188.1	Alternative oxidase 1a
67	AK325031.1	DnaJ-like protein
68	AK321605.1	Vacuolar processing enzyme-1b
69	NM_001313930	Solanum lycopersicum AGAMOUS-like, TAGL1 transcription factor
70	BT012714.1	Pectate lyase
71	NM_001247207.1	histidine decarboxylase (hdc)
72	U13681.1	lipoxygenase (LOX)
73	AK323062.1	Putative uncharacterized protein
74	NM_001247945.1	Mannan endo-1,4-beta-mannosidase 4 precursor
75	AK329757.1	Putative carboxyphosphonoenolpyruvate mutase
76	AK319964.1	Putative uncharacterized protein
77	AK326014.1	Unnamed protein
78	AB061257.1	pyruvate decarboxylase
79	AK326008.1	Cysteine protease
80	AK326536.1	Serine carboxypeptidase-like 11 precursor
81	AK321159.1	Putative uncharacterized protein, Chromosome chr5 scaffold_2
82	AF204784.1	ripening regulated protein DDTFR10/A
83	AK326042.1	Cysteine synthase

84	AK326667.1	Branched-chain amino acid aminotransferase
85	AK325167.1	Putative uncharacterized protein
86	BT012788.1	70 kDa peptidyl-prolyl isomerase
87	NM_001247214.1	heat shock protein (hsp100/ClpB)
88	AK320498.1	Unnamed, similar to polyamine oxidase
89	XM_004253276.1	putative pterin-4-alpha-carbinolamine dehydratase-like
90	BT013418.1	Proline oxidase/dehydrogenase
91	AK319628.1	Adenylate kinase
92	AK329022.1	Peroxiredoxin-2E-1 chloroplast precursor
93	AK325225.1	Proline-rich protein
94	AK247221.1	Putative uncharacterized protein
95	AK322157.1	Ripening regulated protein-like
96	AK320166.1	4-hydroxyphenylpyruvate dioxygenase
97	AK325947.1	Flavonol synthase/flavanone 3-hydroxylase
98	AK319411.1	Glutathione S-transferase
99	NM_001247151.1	enolase (PGH1)
100	AK322168.1	Cathepsin B
101	BT013615.1	Fructose-bisphosphate aldolase
102	AK322999.1	Basic 30 kDa endochitinase precursor
103	AK327343.1	Putative uncharacterized protein
104	NM_001247467.1	Fructokinase 3
105	AK324703.1	similarity to Transcription initiation factor IIE, beta subunit (TFIIE-beta)
106	AK326870.1	hypothetical ABC transporter ATP-binding protein
107	AK325354.1	Non-intrinsic ABC protein 7 chloroplast precursor
108	NM_001246936.1	bZIP transcription factor (ZIP)
109	AK321347.1	Uncharacterized protein
110	NM_001247222.1	Pectinesterase-1 precursor
111	BT014152.1	37kDa chloroplast inner envelope membrane polypeptide
112	X67600.1	heat stress transcription factor
113	AK326094.1	Glucose-6-phosphate 1-dehydrogenase
114	AK319709.1	Putative uncharacterized protein
115	NM_001247541	Xyloglucan endotransglucosylase-hydrolase XTH5
116	AK320858.1	Cullin 1B
117	AK324059.1	Putative spermine synthase
118	BT012996.1	2-oxoglutarate dehydrogenase E2 subunit
119	AK327985.1	SAL1 phosphatase (3'(2'),5'-bisphosphate nucleotidase 1
120	AK325065.1	Amidase-like protein
121	NM_001247112.1	Glutamate decarboxylase
122	NM_001247183.1	Shikimate kinase
123	NM_001247139.1	Farnesyl pyrophosphate synthase
124	BT014044.1	Ribosomal protein L7/L12 C-terminal domain containing protein
125	AK328599.1	Calcineurin B-like protein 1
126	AK324152.1	Putative uncharacterized protein
127	AK246501.1	ACT domain-containing protein

128	U97257.1	Glyceraldehyde 3-phosphate dehydrogenase
129	AK321339.1	Bifunctional lysine-ketoglutarate reductase/saccharopine dehydrogenase

Supplemental Data 2. Sequence of *RIN*, *AP2a/ERF*, *TDR4/FUL1*, *PSY1*, *TAGL1* and *SlNAP7* gene were inserted in TRV2 viral vecter.

Sequence of *PSY1*:

ACAGATGGTCTATGATGTGGTTTTGAGGCAGGCAGCCTTGGTGAAGAGGCAACTGAGATCTACCAATGAGTTAGAAGTGAGCC
GGATAACCTATTCCGGGGAATTTGGGCTTGTTGAGTGAAGCATATGATAGGTGTGGTGAAGTATGTGCAGAGTATGCAAAGACG
TTTAACTTAGGAACTATGCTAATGACTCCCGAGAGAAGAAGGGCTATCTGGGCAATATATGTATGGTGCAGAAGAACAGATGAA
CTTGTTGATGGCCCAAACGCATCATATATTACCCCGGCAGCCTTAGATAGGTGGGAAAATAGGCTAGAAGATGTTTTCAATGGGC
GGCCATTTGACATGCTCGATGGTGCTTTGTCCGATACAGTTTCTAACTTTCCAGTTGATATTCAGCCATTCAGAGATATGATTGAA
GGAATGCGTATGGACTTGAGAAAATCGAGATACAAAAACTTCGACGAACTATACCTTTATTGTTATTATGTTGCTGGTACGGTTG

Sequence of *AP2a/ERF*:

TACATCCGATCTGAACAAGAAGAAGAAGAACCCTAGTAATTACCACTCAAAGCCCTTGAGGTCTAAGTTTATTGACCTTGAAGAT
GAATTTGAAGCTGACTTTCAGCACTTCAAGGATAATTCTGATGATGATGATGATGTGAAGGCATTTGGCCCCAAATCCGTGAGAT
CTGGTGATTCAAACTGCGAAGCTGACAGATCCTCCAAGAGAAAGAGGAAGAATCAGTACCGGGGGATCAGACAGCGTCCTTG
GGGTAAGTGGGCAGCTGAAATACGTGATCCAAGGAAAGGTATTCGAGTCTGGCTTGGTACTTTCAATTCAGCCGAAGAGGCA
GCCAGAGCTTATGATGCTGAGGCGCGAAGGATCAGAGGCAAGAAAGCTAAGGTGAACTTTCCTGATGAAGCTCCAGTGTCTG
TTTC

Sequence of *RIN*:

CTCCTAAAGAAAGCTTATGAACTTTCTATACTTTGTGATGCTGAAATTGCTCTTATTATTTTCTCTAGTCGTGGCAAGCTTTATGAA
TTTTGCAGCAATTCAAGTATGTCCAAGACATTGGAGAGATACCACAGATACAATTATGGTACACTTGAAGGAACCCAAACTTCAT
CAGATTCACAGAACAACTACCAAGAGTATTTGAAGCTTAAAACAAGAGTGGAAATGTTACAACAGTCTCAAAGGCATTTGCTAG
GTGAGGATTTGGGACAATTGGGCACAAAAGACTTGGAACAGCTTGAACGTCAATTGGATTCATCAT

Sequence of *TAGL1*:

GAAGATTGAAATTAAAAGGATCGAAAATACGACAAATCGACAAGTTACGTTCTGCAAGCGTAGAAATGGGCTATTGAAAAAAG
CTTATGAACTTTCTGTTCTTTGTGATGCTGAAGTTTCACTAATTGTATTTTCCAGCCGCGGCCGTCTCTATGAATATGCCAATAACA
GTGTTAGGGCAACTATTGATAGGTACAAGAAACACCATGCTGATTCCACTAGTACTGGATCTGTTTCTGAAGCTAACACTCAGTA
CTACCAGCAAGAAGCATCCAAACTGCGACGACAAATTCGAGATATACAGACTTATAACAGGCAAATAGTTGGAGAGGCATTGG
GCAGTTTAAGCCCTAGAGACCTCAAGAATTTGGAAGGGAAACTTGAAAAGGCCATTGGTAGAGTCCGTTCCAAAAAGAATGA
ATTG

Sequence of *TDR4/FUL1*:

TGATGCTGAGGTTGGTTTGATTGTTTTTTCTACTAAAGGAAAACTCTTTGAATATGCCAACGATTCCTGCATGGAGAGGATACTT
GAAAGATATGAAAGATACTCATTTGCTGAGAAACAGCTTGTTCCTACTGATCATACCTCCCCGGTAAGCTGGACCCTTGAACATG
CAAAACTTAAGGCCAGACTTGAGGTTCTGCAGAGGAACCAAAAGCATTATGTGGGAGAAGATTTGGAGTCCTTAAGTATGAAG
GAACTTCAGAATCTGGAGCACCAGCTTGATTCAGCTCTTAAACACATTCGATCAAGAAAGAATCAATTGATGCATGAGTCCATTT
CTGTGCTTCAAA

Sequence of *SlNAP7*:

AAGCCACCGTCAGCGTCGAATCTCCGTCGTCTTCCTCCACCGACTGCAGGGATGACTCGCCCAAGGTCTTACTTGAAGTCCGGG
ACCTCTCCGCTGTCATAGTCGAGTCAAAGCAGCAAATTCTCAATGGTGTTAACCTCACTGTCCGCCAAGGCGAGGTACATGCTGT
AATGGGTAAGAATGGTTCTGGAAAGAGCACCTTTGCCAAGGTTCTTGTTGGGCATCCAGATTATGAAATTACAGGAGGCAGTG
TGTCATTTAAAGGTGAGAATCTACTTGAGATGGAACCCGAGGAAAGATCTCTTGCTGGTCTTTTCATGAGCTTCCAGTCCCCAG
TTGCCATACCTGGAGTAAGCAATATTGACTTTCTTAACATGGCGTATAATGCTCAAAGGAGGAACCTTGGACTGCCAGAACTGG
GACCAATTGAGTTTTACGGGTACATTGCCCCGAAGCTTGAACTTGTCAACATGAAGATAGACTTCTTGAACAGAAATGTAAATG
AAGGATTCAGTGGTGGAGAAAGGAAACGCAATGAGATTCTGCAACTAGCGGTTCTCGG

Supplemental Data 3: The coding sequence alignment of *Slnap7* and *Atnap7*.

```
Alignment of SlNAP7_CDS(upper line) and AtNAP7_CDS(lower line)
Similarity=62.16%(777/1250)              Identity=70.51%(660/936)
Gap=8.24%(84/1020)
-----------------
1     .......................................ATGGCTGTTTTCAGTCCCACT
                                             || |||   |||  ||| |||
1     ATGGCCGGCGTTAACCTACAGCTCCGTCACGCATACTCCATAGCTCAATTCGTTCCAACT

22    TGTTCTTCTACTTCCCCACTTCTCTCCTTCAGAAACAACATCTCTTTCTTCCCTTCACAT
        ||||||| || | || ||||   |     ||     | ||      |  || || | |
61    GTTTCTTCTCCTCCTCCTCTTCCTACTCAGCGAGTTCGCCTCGGGACTTCACCCTCTCGT

82    CGCCCTTCTTT...TCCCGGAG.....TCCGGTGC.ACTCTCACAGTGAAAGCCACC...
         |  |  |    || | |||     ||||  ||  |||  |   ||  | | ||
121   GTTCTATTATGCAATCTCCGAGCTAATTCCGCCGCCGCTCCAATCCTGCGAACTACTCGC

130   ......GTCAGCGTC...GAATCTCCGTCGTCTTCCTCCACCGACT..............
            || |  ||    |  ||| |    || ||  ||  ||| |
181   CGTTCAGTTATTGTTTCGGCGTCTTCCGTATCCTCAGCCGTCGATTCGGATTCGTTGGTG

167   ....GCAGGGATGACTCGCCCAAGGTC...TTACTTGAAGTCCGGGACCTCTCCGCTGTC
             | ||||||  |   | | ||   || ||||||||  ||||  |    || ||
241   GAAGATCGTGATGACGTGGGGAGGATCCCGTTGCTTGAAGTTAGGGATTTGAGAGCAGTG

220   ATAGTCGAGTCAAAGCAGCAAATTCTCAATGGTGTTAACCTCACTGTCCGCCAAGGCGAG
      || |  || || |  ||  | || || || || || ||  |   ||||  | |||| |||
301   ATTGCTGAATCGAGACAAGAGATACTGAAAGGCGTCAATTTGGTTGTCTACGAAGGAGAG

280   GTACATGCTGTAATGGGTAAGAATGGTTCTGGAAAGAGCACCTTTGCCAAGGTTCTTGTT
      || || || || ||||| ||||| ||||| ||||||||||| ||| | ||||||||||||
361   GTTCACGCAGTGATGGGGAAGAACGGTTCAGGAAAGAGCACGTTTTCGAAGGTTCTTGTT

340   GGGCATCCAGATTATGAAATTACAGGAGGCAGTGTGTCATTTAAAGGTGAGAATCTACTT
      || ||||| ||||||||| | || ||||| ||| |    ||||||||  |||||||||||
421   GGTCATCCCGATTATGAAGTGACGGGAGGGAGTATTGTGTTTAAAGGGCAGAATCTACTT

400   GAGATGGAACCCGAGGAAAGATCTCTTGCTGGTCTTTTCATGAGCTTCCAGTCCCCAGTT
      || |||||||| ||||| ||||||||||| ||||| || ||||| |||||||||||||||
481   GATATGGAACCAGAGGATAGATCTCTTGCCGGTCTCTTTATGAGTTTCCAGTCCCCAGTT

460   GCCATACCTGGAGTAAGCAATATTGACTTTCTTAACATGGCGTATAATGCTCAAAGGAGG
      |  || ||||| || |||||||| || ||  | || ||||| |  |||||||  |  ||
```

```
541   GAGATCCCTGGTGTTAGCAATATGGATTTCTTGAATATGGCATTCAATGCTCGGAAAAGA

520   AAACTTGGACTGCCAGAACTGGGACCAATTGAGTTTTACGGGTACATTGCCCCGAAGCTT
      || ||||| | |||||| || |  |||||  |||| ||| |  || | |   | || ||
601   AAGCTTGGTCAGCCAGAGCTTGATCCAATCCAGTTCTACAGCCACTTGGTATCAAAACTC

580   GAACTTGTCAACATGAAGATAGACTTCTTGAACAGAAATGTAAATGAAGGATTCAGTGGT
      ||| |||| || |||||||  || ||  | ||||||||||| ||||||||||| ||||||
661   GAAGTTGTGAATATGAAGACCGATTTTCTCAACAGAAATGTCAATGAAGGATTTAGTGGT

640   GGAGAAAGGAAACGCAATGAGATTCTGCAACTAGCGGTTCTCGGGGCTGACTTGGCAATA
      || |||||||||||||||||||||| ||  ||||||| || || ||||| ||||| |||
721   GGTGAAAGGAAACGCAATGAGATTCTACAGTTAGCGGTCCTTGGAGCTGAGTTGGCTATA

700   CTGGATGAGATTGATTCTGGTTTAGATGTTGACGCACTTCGAGACGTAGCAAAGGCAGTA
      ||||| ||||||||||| || || |||||||| || |||| ||| || |||||||| ||
781   CTGGACGAGATTGATTCAGGGTTGGATGTTGATGCTCTTCAAGATGTGGCAAAGGCGGTG

760   AATGGACTTCTATCGCCAAAGAATTCAGTGTTGATGATTACTCATTACTTACGATTATTA
      || || ||| |  | ||||| || || ||  ||||||| || || |||  |||  || |
841   AACGGGCTTTTGACACCAAAAAACTCTGTTCTGATGATAACGCACTACCAACGCCTACTT

820   GAATTCATCAAGCCGACGTATATCCATATCATGGAGAAAGGGAGAATCGTGAAGACTGGA
      || | |||||| || ||    || |||||||||||||| ||||||||| | || || |||
901   GACTACATCAAACCAACTCTCATACATATCATGGAGAATGGGAGAATCATTAAAACCGGA

880   GACATATCCATAGCTAAAGTTCTGGAGAAAGAAGGGTACAAAGCAATTTCTGGCCCATAG
      ||||  ||| | || ||| | ||||| || ||||| |||||||| || || ||
961   GACAACTCCTTGGCCAAACTACTGGAAAAGGAAGGCTACAAAGCGATATCCGGTTAG...
```

Supplemental Data S4. All gene-specific primers used for expression analysis by semi RT-PCR and real-time quantitative PCR.

Gene	Acc no.	Primer name	Primer sequences (5'-3')
SlNAP7	XM_004240794.1	NAP7 semi RT-PCR F	TTCTACTTCCCCACTTCTCT
		NAP7 semi RT-PCR R	TCCTTCATTTACATTTCTGT
SlNAP7	XM_004240794.1	NAP7 QPCR F	CTCTCCGCTGTCATAGTC
		NAP7 QPCR R	TCTCAAGTAGATTCTCACCTT
ACTIN	AB199316-1	Actin semi-RT F	GTCGTGACCTTACTGATA
		Actin semi-RT R	AGACACTGTACTTCCTCT
ACTIN	AB199316-1	Actin QRT-PCR F	CAGCAGATGTGGATCTCAAA
		Actin QRT-PCR R	CTGTGGACAATGGAAGGAC
HEMA2	NM_100868.2	HEMA2 QRT-PCR F	CGTGCTCTTCCGATGAACAATA
		HEMA2 QRT-PCR R	ATCACCACAATGCTGCTTCTT
ChlH	XM_004236562.1	chl H QRT-PCR F	GGAACGACGAGAAGCAACTT
		chl H QRT-PCR R	CAGCACCAGGAGCATCAC
CHLM	XM_004235797.1	CHLM QRT-PCR F	TCCACCGCTACCGACATC
		CHLM QRT-PCR R	CCTCCTCCGCCTGAAGTT
CHLG	XM_004246270.1	CHLG QRT-PCR F	TCCTCAACACTGTGCCATCTA
		CHLG QRT-PCR R	TTGCTCTAACAACGAGTCTTCTTC
HEMF	XM_004248364.1	HEMF QRT-PCR F	TGTGCCAATTCCGTCATTCC
		HEMF QRT-PCR R	CTCTCAATCCTACCTCCTGTCTT
HEMC	XM_004243933.1	HEMC QRT-PCR F	TTCTTCGCCTTGCCGTAA
		HEMC QRT-PCR R	TCGTGTTCCAACTCTGATGAC
HEME1	XM_004240802.1	HEME1 QRT-PCR F	ATCGTCTTCTTCTTCTCGCTCTAA
		HEME1 QRT-PCR R	CCGAACAGCATCAAGCAACA
DVR	XM_006342717.1	DVR QRT-PCR F	CTTAGGAGGTCAGGTTGAATTGG
		DVR QRT-PCR R	TCTTGTTCGCTTATCGGCTTG
Chl I	XM_004248091.1	chl I QRT-PCR F	CGGGCTTTCTTCTGTTACGATT
		chl I QRT-PCR R	AATGCTCTTGCTGCTCTGTTAG
GSA1	NM_001247761.1	GSA1 QRT-PCR F	GCTTGCCAGTAGGTGCTTA
		GSA1 QRT-PCR R	CCTGTCCTTGTAACCGCTTAA
CHL	XM_004250263.1	CHL QRT-PCR F	TTCTACGCACCTTCATCAACTG
		CHL QRT-PCR R	CAAGCACAAGCCGTAAGTCT
CRD1	XM_004249051.1	CRD1 QRT-PCR F	TGGACAGGATGGTGGAGATTA
		CRD1 QRT-PCR R	TACGCAGCCAATAACTCAGAAG
PrO	XM_004229317.1	PrO QRT-PCR F	TCATCCTCATCGCCGTCAT
		PrO QRT-PCR R	CCGTGTTCTCCAGCCATTG
HEMA1	XM_004237988.1	HEMA1 QRT-PCR F	CAAGTCAAGCAAGTAGTCAAGGT
		HEMA1 QRT-PCR R	GCAGTAGTGTGGCAGGATTC
MEMD	XM_004238133.1	MEMD QRT-PCR F	CTCGGCTGAACACTTACACAA
		MEMD QRT-PCR R	AGAAGGAGATGCTACAGTAACAAC
PORA	XM_004248225.1	POR1 QRT-PCR F	CGAGATTGTTGCTTGATGACTTG
		POR1 QRT-PCR R	CCAGCCAGAGTATTCGTGTTC

POR	XM_004251804.1	PORL1 QRT-PCR F	TCCATAGACGATACCACGAAGAG
		POR L1 QRT-PCR R	TCCCTGAATAGCCCTGTTGT
ChlD	XM_004236579.1	ChlD QRT-PCR F	CGGGCTTTCTTCTGTTACGATT
		ChlD QRT-PCR R	AATGCTCTTGCTGCTCTGTTAG
HEMB1	XM_004245253.1	HEMB1 QRT-PCR F	GGTCGTCAGCAGCAAGAG
		HEMB1 QRT-PCR R	TCCATCCAAGCCTATAACATCCA
GGPPS1	NM_001247158.1	GGPS1 QRT-PCR F	GCTTCTTGTGAAGTTGTAGGA
		GGPS1 QRT-PCR R	GGACGAGTGACATGGTATG
GGPPS2	NM_001247373.1	GGPS2 QRT-PCR F	CTGCCTGTGAACTTGTTG
		GGPS2 QRT-PCR R	AGATCGTCGTCATCCATAC
LCY-E	XM_006353482.1	LCY-e QRT-PCR F	AACTGATGCTACGATTGAAC
		LCY-e QRT-PCR R	ATCTGACGACTGAATAACCT
PDS	NM_001247166.1	PDS QRT-PCR F	CATTGATTATCCAAGACCAGAG
		PDS QRT-PCR R	CCAGCAATAACAATCTCCAA
PSY1	EF157836.1	PSY1 QRT-PCR F	ATGTCTGTTGCCTTGTTATG
		PSY1 QRT-PCR R	TTCCACCACCTCTATTGATT
PSY2	NM_001247742.1	PSY2 QRT-PCR F	CAGTAGGATTGATGAGTGTTC
		PSY2 QRT-PCR R	CTCTGAGTATATTGGTTAGTTGAT
ZDS	NM_001247454.1	ZDS QRT-PCR F	TCTTGCTGGCTCATATACA
		ZDS QRT-PCR R	AGACTCAACTCATCAGATAGG
LCY-B	XM_006364371.1	LCY-beta qRT-PCR-F	CCTGGCTTGCGTATAGAT
		LCY-beta qRT-PCR-R	CTTCTTCAATGCTCTTCACTT
DXS	NM_001247743.2	qPCR-DXS-F	GCGGAGCTATTTCACATGGT
		qPCR-DXS-R	CTGCTGAGCATCCCAAT
DXR	NM_001247624.2	qPCR-DXR-F	ACCATCATCTGGTTTGAGTCCAG
		qPCR-DXR-R	GTGATGATGTAGTACATGTAACC
CRTISO	NM_001309231.1	qPCR-CRTISO-F	TTTTGGCGGAATCAACTACC
		qPCR-CRTISO-R	GAAAGCTTCACTCCCACAGC
CYC-B	NM_001247516.2	qPCR-CYC-B-F	TGTTATTGAGGAAGAGAAATGTGTGAT
		qPCR-CYC-B-R	TCCCACCAATAGCCATAACATTTT

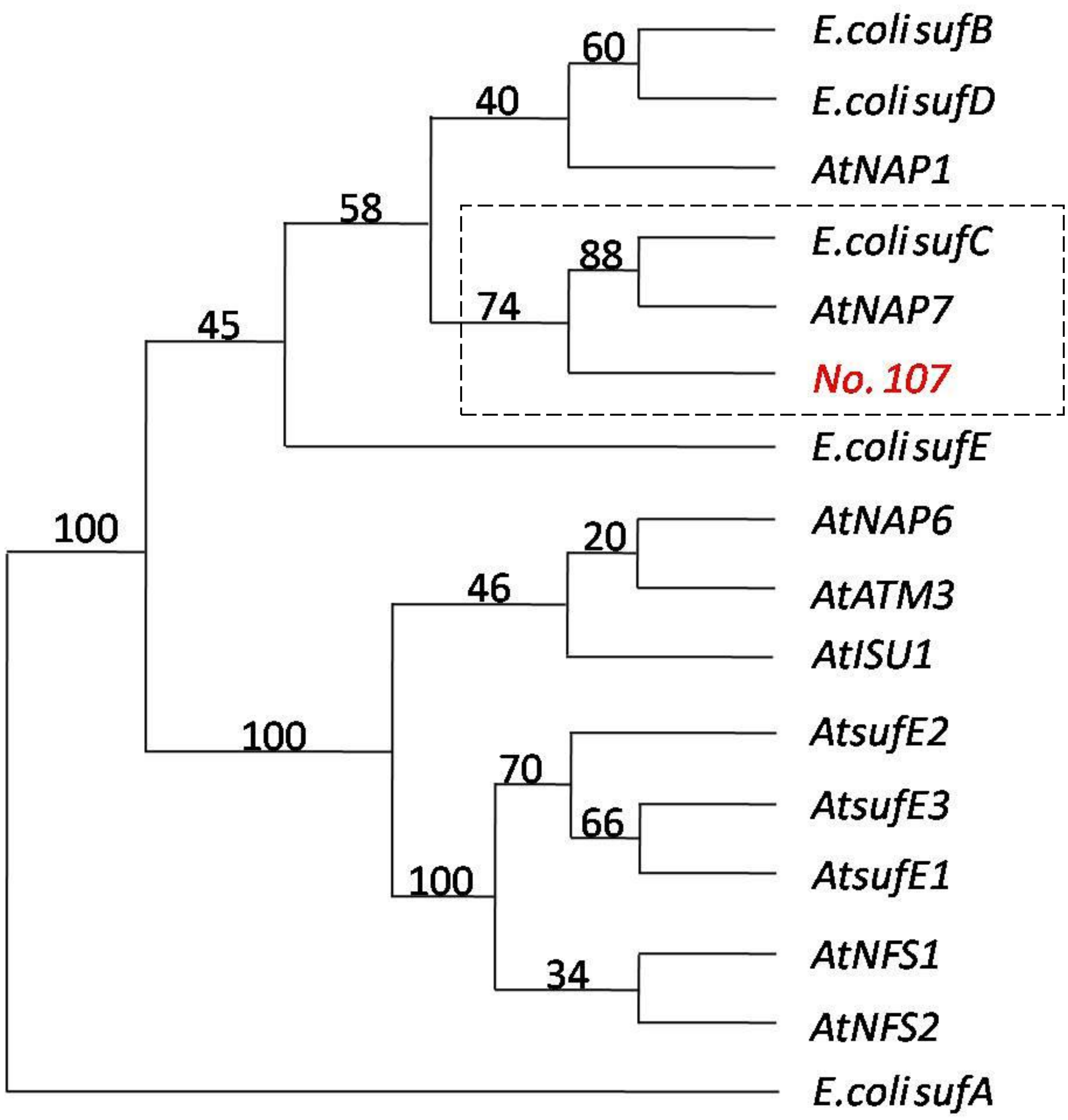

Figure S1: The strict consensus tree for 16 Fe-S assembly relative genes in *E. coli*, *Arabidopsis* and *Solanum lycopersicum*. The results showed the *No.107*, *AtNAP7* and *E. coli sufC* were clustered into one clade.
